# Creation and evaluation of a fidelity measure of collaborative care for maternal depression in Federally Qualified Health Centers

**DOI:** 10.1186/1748-5908-10-S1-A71

**Published:** 2015-08-20

**Authors:** Ian M Bennett, Ya-Fen Chan, Johnny Mao, Jurgen Unutzer, Enola Proctor

**Affiliations:** 1 Family Medicine and Community Health, Perelman School of Medicine of the University of Pennsylvania, Philadelphia, PA, USA; 2 University of Washington School of Medicine, Seattle, WA, USA; 3 George Warren Brown School of Social Work, Washington University in St. Louis, St. Louis, MO, USA

## Objective

To develop and evaluate a fidelity measure of the evidence based collaborative care model for maternal depression care in multidisciplinary primary care sites caring for low income and race/ethnic minority populations.

## Method

Fourteen federally qualified community health centers (FQHCs) in Seattle and King County, Washington participated (2008-2015) in an effort to implement collaborative care for depression in high risk mothers. An expert panel based framework was used to identify key elements of the collaborative care model. This theory based framework guided operationalization of fidelity measures of this intervention from quantitative metrics from an electronic clinical care management and health record (CMTS) used by all of the sites.

## Findings

1748 of 2500 (70%) unique women who received care for depression symptomatology over the course of the study period were eligible for inclusion in this analysis. Six fidelity measures were created from the CMTS database covering elements such as the use of a patient registry, evidence based measures, and appropriate clinical follow up. A simple dichotomous score for each element was associated with increased clinical improvement in depression for four of five of the measures after adjusting for age, pregnancy status, race/ethnicity, baseline depression and anxiety symptoms, and suicidal ideation (aOR 1.44-3.04; p < 0.001). A summary measure of these scores had a higher point estimate of clinical benefit than any individual item (aOR 7.41, 95% CI 4.72-11.62) supporting our hypothesis that these reflect distinct components of a summary scale.

## Summary

A fidelity measure based on a theoretical framework and constructed from existing clinical data was predictive of clinical outcomes. Additional work is needed to determine if these measures are useful for monitoring implementation success of the collaborative care model.

## Funding

Public Health Seattle & King County, Eitel Family Foundation (Unutzer), Agency for Healthcare Research and Quality (Bennett), University of Pennsylvania Implementation Science Workgroup (Bennett).

